# Support Vector Machines in Polymer Science: A Review

**DOI:** 10.3390/polym17040491

**Published:** 2025-02-13

**Authors:** Ivan Malashin, Vadim Tynchenko, Andrei Gantimurov, Vladimir Nelyub, Aleksei Borodulin

**Affiliations:** 1 Artificial Intelligence Technology Scientific and Education Center, Bauman Moscow State Technical University, 105005 Moscow, Russia; 2 Scientific Department, Far Eastern Federal University, 690922 Vladivostok, Russia

**Keywords:** support vector machines, support vector regression, predictive analytics, polymer properties

## Abstract

Polymer science, a discipline focusing on the synthesis, characterization, and application of macromolecules, has increasingly benefited from the adoption of machine learning (ML) techniques. Among these, Support Vector Machines (SVMs) stand out for their ability to handle nonlinear relationships and high-dimensional datasets, which are common in polymer research. This review explores the diverse applications of SVM in polymer science. Key examples include the prediction of mechanical and thermal properties, optimization of polymerization processes, and modeling of degradation mechanisms. The advantages of SVM are contrasted with its challenges, including computational cost, data dependency, and the need for hyperparameter tuning. Future opportunities, such as the development of polymer-specific kernels and integration with real-time manufacturing systems, are also discussed.

## 1. Introduction

The integration of machine learning (ML) in material science, particularly polymer science, has become increasingly prevalent in recent years. Polymers, with their diverse properties and complex molecular structures, pose challenges in terms of prediction [1], optimization [2,3], and analysis [4,5]. Traditional methods often fall short when it comes to handling the vast amount of data generated in polymer research. As a result, ML approaches, including Support Vector Machines (SVMs), have gained considerable attention for their potential to enhance polymer science.

SVM is a supervised learning algorithm used for classification, regression, and outlier detection [6]. It has shown promise in dealing with high-dimensional and nonlinear data, which are common in the study of polymers. This review aims to explore the applications of SVM in polymer science, discuss its advantages and limitations, and offer insights into future trends in the field.

**Figure 1:**
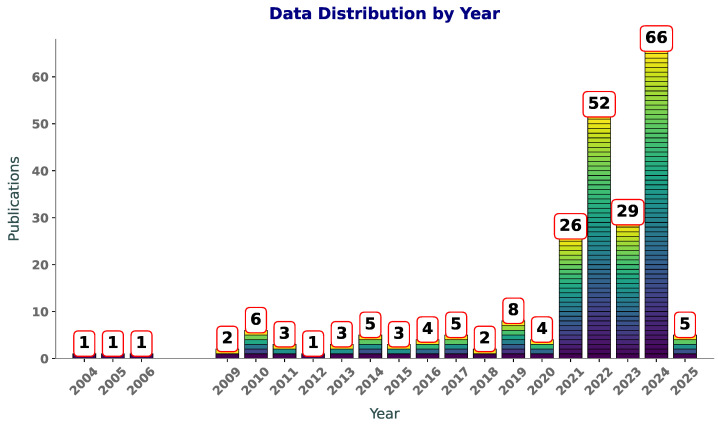
Year-wise distribution of publications featuring “SVR”, “SVM”, and “polymers” in title or abstract.

Figure 1 illustrates the number of publications per year on Scopus with the terms “SVM”, “SVR”, and “polymers” in the title or abstract. Between 2004 and 2009, the publication counts are sparse, with only one paper published per year in most cases. This suggests that the application of SVM andSVR to polymers was in its infancy during this period, likely due to limited awareness of ML methods in the field or challenges in accessing computational tools. From 2010 to 2019, there is a slight increase in activity, with publication counts fluctuating between one and eight per year. This phase likely reflects an exploratory period, where researchers began to experiment with these techniques but had not yet widely adopted them in polymer research.

An increase in publications is observed post-2019, starting with a modest rise in 2020 and reaching a peak of 66 publications in 2024. This sharp growth aligns with advancements in computational techniques and the increasing availability of ML libraries and frameworks, making these tools more accessible to researchers. Additionally, the growth of polymer informatics as an interdisciplinary field has likely contributed to this surge, as researchers use SVM and SVR to model and predict polymer properties. The increasing number of publications reflects a rising awareness of the benefits of these techniques. As computational resources continue to improve and interdisciplinary collaborations expand, it is likely that SVM and SVR will remain integral to polymer research, driving innovations in material design and property prediction.

**Figure 2:**
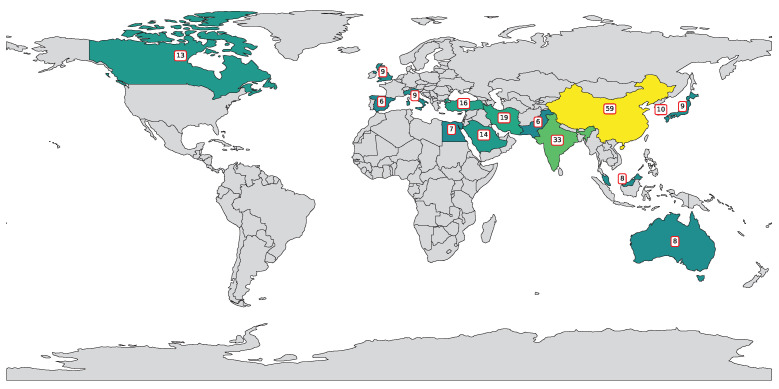
Country-wise distribution of publications featuring “SVR”, “SVM”, and “polymers” in title or abstract.

Figure 2 highlights a global effort in applying SVM and SVR to polymer research, led by China (59 publications), followed by India (33) and the United States (31), reflecting strong investments in interdisciplinary research. Iran (19), Turkey (16), and Saudi Arabia (14) show regional contributions, while countries like South Korea, Italy, Japan, and the UK (9–10 each) indicate established interest. Emerging contributors include Australia, Malaysia, and Egypt (7–8), with many nations producing 1–2 publications, demonstrating the growing global adoption of ML in polymer science.

**Figure 3:**
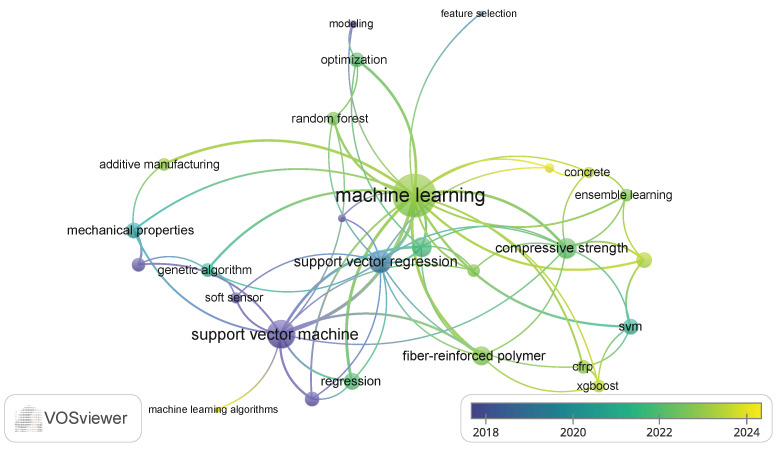
Keyword co-occurrence map based on VOSviewer analysis.

The keyword occurrence map (Figure 3) reflects diverse and interdisciplinary research areas, emphasizing the integration of ML and advanced modeling techniques with polymer science, materials engineering, and sustainability studies. Topics like infrared spectroscopy, chemometrics, and regression highlight analytical and predictive modeling applications. Keywords such as fly ash, geopolymer, and sustainability emphasize green materials and environmental considerations.

The inclusion of polymer composites, fiber-reinforced polymer (FRP), and mechanical properties indicates a focus on structural materials, while 3D printing, process control, and electrospinning show interest in advanced manufacturing techniques. SVR is dominant in predictive modeling, often used for optimizing properties such as compressive strength and bond strength.

## 2. SVM Outlook

ML has become a cornerstone of artificial intelligence, with its ability to enable systems to improve performance through experience rather than explicit programming. Among various ML algorithms, SVMs stand out as a robust and well-established supervised learning technique. Developed in the early 1990s by Vladimir Vapnik and his colleagues [7,8], SVMs were initially rooted in statistical learning theory. The algorithm was designed to find an optimal hyperplane that separates data into classes with the maximum margin, ensuring both accuracy and generalization.

The original formulation of SVMs was limited to linear classification. However, the introduction of kernel methods significantly expanded their capabilities, allowing for nonlinear classification by transforming input data into higher-dimensional spaces. This breakthrough enabled SVMs to handle complex, nonlinearly separable datasets effectively. Over time, SVMs have been applied to a wide range of tasks, including image recognition, text categorization, and bioinformatics.

In polymer science, SVMs began gaining attention in the early 2000s for their ability to model complex relationships between polymer properties and experimental parameters. Their versatility in handling small datasets with high-dimensional features made them suitable for this domain. Over the past two decades, SVMs have been employed to classify polymer types, predict mechanical and thermal properties, and optimize processing parameters.

Despite their widespread adoption, SVMs face challenges in terms of computational scalability with large datasets and interpretability when compared to more recent algorithms [9], such as neural networks. Nonetheless, their theoretical foundations, performance on structured data, and ability to generalize from limited samples continue to make them a valuable tool in polymer analysis.

### 2.1. Principles of SVM

SVM is a supervised learning algorithm primarily used for classification and regression tasks. The main idea of SVM is to find the optimal hyperplane that separates data points of different classes in a high-dimensional feature space.

Consider a binary classification problem, where the data points $x_{1},x_{2},\ldots ,x_{n}$ belong to one of two classes, denoted as +1 or −1. The task of SVM is to find a hyperplane that divides these two classes with the maximum margin.

For a linear classifier, the decision boundary can be represented by the equation of a hyperplane:

(1) $$w^{T}x+b=0$$

where

- *w* is the weight vector perpendicular to the hyperplane;
- *x* is the input feature vector;
- *b* is the bias term (offset from the origin).

The goal is to find the optimal values of *w* and *b* such that the hyperplane maximizes the margin between the data points of the two classes.

The margin is defined as the distance between the hyperplane and the closest data points (the support vectors). To maximize this margin, we seek to minimize the following objective function:

(2) $$\mathrm{Minimize}\;\frac{1}{2}{∥w∥}^{2}$$

This formulation is subject to the following constraints for each data point $x_{i}$:

(3) $$y_{i}\left(w^{T}x_{i}+b\right)\geq 1,\;\forall i=1,2,\ldots ,n$$

where $y_{i}\in \left\{+1,-1\right\}$ represents the class label of the data point $x_{i}$.

The optimization problem is formulated as follows:

(4) $$\min_{w,b}\frac{1}{2}{∥w∥}^{2}$$

subject to the following constraints:

(5) $$y_{i}\left(w^{T}x_{i}+b\right)\geq 1,\;\forall i$$

This is a convex optimization problem, and can be solved using Lagrange multipliers. The Lagrangian for this problem is as follows:

(6) $$L\left(w,b,\lambda \right)=\frac{1}{2}{∥w∥}^{2}-\sum_{i=1}^{n}\lambda_{i}\left[y_{i}\left(w^{T}x_{i}+b\right)-1\right]$$

where $\lambda_{i}$ are the Lagrange multipliers associated with each constraint. The KKT (Karush–Kuhn–Tucker) conditions are used to find the optimal values of *w*, *b*, and $\lambda_{i}$.

By solving this optimization problem, we obtain the optimal weight vector $w^{*}$ and bias $b^{*}$ that define the decision boundary. The classification rule for a new input *x* is given by

(7) $$f\left(x\right)=\mathrm{sign}\left(w^{T}x+b\right)$$

where the sign function determines whether the point *x* belongs to the positive or negative class.

**Figure 4:**
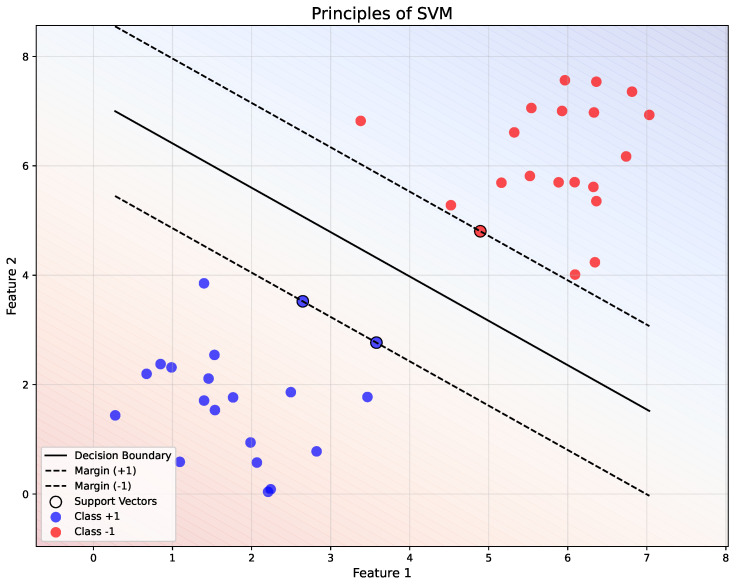
Illustration of the principles of SVM.

Figure 4 demonstrates the separation of two classes (+1 and −1) using a linear decision boundary ($w^{T}x+b=0$, solid black line). The margins (+1 and −1) are represented by dashed black lines, with the support vectors (highlighted points) lying on these margins. The background gradient indicates the decision regions, showing the classification boundaries in the feature space.

**Figure 5:**
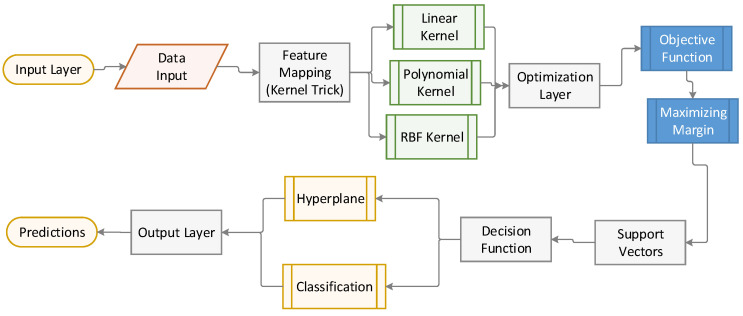
Illustration of the architecture of SVM.

Figure 5 illustrates the inner workings of the SVM model, from the input layer (data) to the output (classification), with a focus on the role of support vectors and the application of kernel methods.

### 2.2. Nonlinear Classification with Kernels

In many real-world problems, the data are not linearly separable. To address this, SVM uses kernel functions to map the input data into a higher-dimensional feature space where a linear separation is possible.

The kernel trick is based on the idea of applying a kernel function $K(x_{i},x_{j})$ that computes the inner product $\langle \phi \left(x_{i}\right),\phi \left(x_{j}\right)\rangle$ in a higher-dimensional space H, without explicitly computing the transformation $\phi (x_{i})$.

For example, common kernel functions include the following:

1. The linear kernel [10] $K\left(x_{i},x_{j}\right)=x_{i}^{T}x_{j}$ is the simplest kernel function, representing the dot product between two vectors $x_{i}$ and $x_{j}$. It is equivalent to using no kernel and is primarily used when the data is linearly separable in the input space. This kernel is computationally efficient and is often applied in text classification problems like document categorization or spam filtering, where the data features are high-dimensional but inherently linear.
2. The polynomial kernel [11] $K\left(x_{i},x_{j}\right)={\left(x_{i}^{T}x_{j}+c\right)}^{d}$ extends the linear kernel by introducing nonlinear interaction terms through the degree *d* of the polynomial. The constant *c* controls the influence of higher-order terms. This kernel is effective for problems where interactions between features are significant, such as image recognition or pattern analysis tasks, especially when the relationship between features is not strictly linear but not highly complex either.
3. The radial basis function (RBF) kernel [12]—$K\left(x_{i},x_{j}\right)=\exp\left(-\frac{∥x_{i}-x_{j}{∥}^{2}}{2\sigma^{2}}\right)$, also known as the Gaussian kernel, is one of the most widely used kernels. It maps the input data into an infinite-dimensional feature space. The parameter σ controls the spread of the Gaussian function, determining the influence of a single data point. The RBF is effective when the data are not linearly separable and have complex, nonlinear relationships. It is used extensively in fields such as bioinformatics, image processing, and anomaly detection.
4. The Sigmoid kernel [13] $K\left(x_{i},x_{j}\right)=\tanh\left(\alpha x_{i}^{T}x_{j}+c\right)$ resembles the activation function of a neural network. Parameters α and *c* control the shape of the kernel. It is suitable for problems where data relationships mimic those modeled by neural networks. While less popular than the RBF kernel, it is used in applications such as text classification or recommendation systems.
5. The Laplacian kernel [14] $K\left(x_{i},x_{j}\right)=\exp\left(-\frac{∥x_{i}-x_{j}∥}{\sigma }\right)$ is similar to the RBF kernel but uses the $L_{1}$-norm instead of the $L_{2}$-norm in the distance calculation. It is more robust to outliers than the RBF kernel and is applied in time-series analysis or problems where the data have a natural sparsity.
6. The exponential kernel [15]—$K\left(x_{i},x_{j}\right)=\exp\left(-\frac{∥x_{i}-x_{j}∥}{\sigma }\right)$ is a special case of the RBF kernel where the distance is measured exponentially. It is used in signal processing and spatial analysis when the influence of a point decays sharply with distance.
7. The rational quadratic kernel [16]—$K\left(x_{i},x_{j}\right)=1-\frac{∥x_{i}-x_{j}{∥}^{2}}{∥x_{i}-x_{j}{∥}^{2}+c}$ acts as a weighted sum of RBF kernels with different bandwidths. It is used in problems where the data have varying degrees of similarity over different scales, such as geostatistics or environmental modeling.
8. Wavelet kernels [17] $K\left(x_{i},x_{j}\right)=\prod_{k=1}^{d}\cos\left(\frac{x_{i,k}-x_{j,k}}{\sigma }\right)\exp\left(-\frac{∥x_{i}-x_{j}{∥}^{2}}{2\sigma^{2}}\right)$ are suitable for capturing both the frequency and localization characteristics of data. They are used in applications involving signal and image processing, where localized patterns or features are important.
9. String kernels [18] $K\left(x_{i},x_{j}\right)=\sum_{s\in S}w\left(s\right)\mathrm{occ}\left(s,x_{i}\right)\cdot \mathrm{occ}\left(s,x_{j}\right)$ compare the similarity of sequences based on the occurrence of substrings *s* from a set *S*, weighted by w(s). These are used in bioinformatics for DNA and protein sequence analysis or in natural language processing for text similarity.

**Figure 6:**
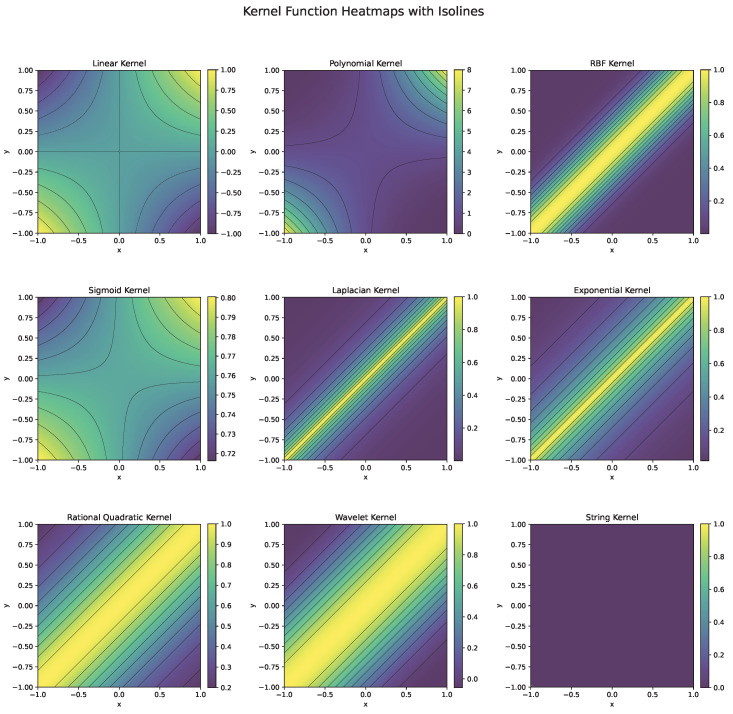
Heatmaps and isolines of kernel functions used in SVM.

Each kernel function (Figure 6) is chosen based on the nature of the data and the problem at hand. For instance, the RBF kernel is preferred for complex, nonlinear problems, while the linear kernel is effective for high-dimensional, linearly separable data. Polynomial kernels work well when feature interactions matter, and specialized kernels like string or wavelet kernels cater to domain-specific tasks.

The SVM optimization problem remains similar, but instead of directly computing the dot product $w^{T}x_{i}$, we compute the kernel $K(x_{i},x_{j})$. The optimization problem becomes

(8) $$\min_{w,b}\frac{1}{2}\sum_{i,j}\alpha_{i}\alpha_{j}y_{i}y_{j}K\left(x_{i},x_{j}\right)-\sum_{i}\alpha_{i}$$

where $\alpha_{i}$ are the Lagrange multipliers corresponding to each training point.

### 2.3. SVM for Regression

SVM can also be used for regression tasks, known as Support Vector Regression (SVR) [19,20]. In SVR, the goal is to find a function that approximates the relationship between input *x* and output *y*, where the data points are not strictly linear.

The SVR model aims to fit the data within a margin of tolerance ϵ, and the objective is to minimize the error outside this margin. The optimization problem for SVR is as follows:

(9) $$\min_{w,b,\xi ,\xi^{*}}\frac{1}{2}{∥w∥}^{2}+C\sum_{i=1}^{n}\left(\xi_{i}+\xi_{i}^{*}\right)$$

Subject to the following constraints:

(10) $$y_{i}-\left(w^{T}x_{i}+b\right)\leq \epsilon +\xi_{i},\;\left(w^{T}x_{i}+b\right)-y_{i}\leq \epsilon +\xi_{i}^{*}\;$$

where

- $\xi_{i}$ and $\xi_{i}^{*}$ are slack variables that allow for deviations from the margin;
- *C* is a regularization parameter that controls the trade-off between maximizing the margin and minimizing the error.

**Figure 7:**
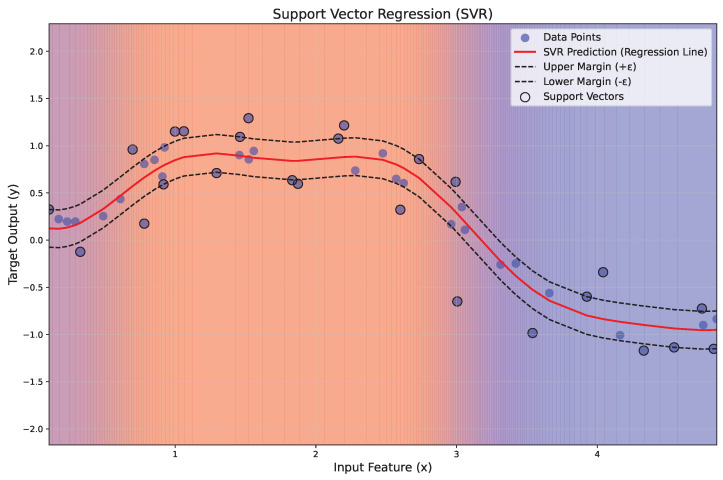
Illustration of the principles of SVR.

Figure 7 illustrates the application of SVR to a nonlinear regression problem. The red line represents the regression function $w^{T}x+b$, while the dashed black lines indicate the margin of tolerance (±ε). Data points within the margin are ignored, while those lying on or outside the margin, termed support vectors, are highlighted with black circles. A gradient background visually represents the regression regions, transitioning from blue to red with transparency for esthetic clarity.

### 2.4. SVM Optimization Using Dual Formulation

The dual formulation of the SVM optimization [21] problem is often used because it simplifies the problem and makes it easier to compute kernel functions. The dual form of the problem is as follows:

(11) $$\max_{\alpha }\left(\sum_{i=1}^{n}\alpha_{i}-\frac{1}{2}\sum_{i,j=1}^{n}\alpha_{i}\alpha_{j}y_{i}y_{j}K\left(x_{i},x_{j}\right)\right)$$

subject to

(12) $$0\leq \alpha_{i}\leq C,\;\sum_{i=1}^{n}\alpha_{i}y_{i}=0$$

where $\alpha_{i}$ are the Lagrange multipliers. Once the dual problem is solved, the optimal hyperplane parameters can be computed.

### 2.5. SVM Workflow in Polymer Science

**Figure 8:**
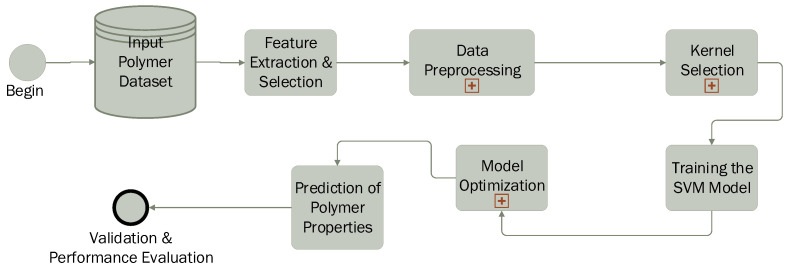
SVM pipeline in polymer science.

The application of the SVM in polymer science follows a structured workflow (Figure 8), enabling the prediction and classification of polymer properties based on experimental or computational data. The process begins with data collection, where measured or calculated polymer characteristics such as molecular weight, crystallinity, glass transition temperature ($T_{g}$), melting temperature ($T_{m}$), viscosity, and mechanical properties are gathered. Subsequently, the most relevant features influencing polymer behavior are selected to improve model efficiency.

The next step involves data preprocessing, including normalization and scaling, to ensure consistency and prevent biases caused by differences in feature magnitudes. A crucial aspect of the process is kernel selection: a linear kernel is used when data are linearly separable, while nonlinear kernels such as the radial basis function (RBF) or polynomial kernel are applied to capture complex molecular interactions.

The SVM model is then trained using labeled data, where it learns to distinguish between polymer classes (classification) or predict specific properties (regression). To enhance accuracy, model optimization is performed by fine-tuning parameters such as the regularization parameter (*C*) and kernel-specific hyperparameters (e.g., gamma for the RBF kernel). Cross-validation techniques, such as k-fold validation, are employed to prevent overfitting. Once trained, the model is used to predict unknown polymer properties, including thermal stability, mechanical strength, biodegradability, and solubility.

In the final stage, the predicted results are compared with experimental data to assess model accuracy. Performance evaluation metrics such as mean absolute error (MAE), root mean square error (RMSE), coefficient of determination ($R^{2}$), and confusion matrices (for classification tasks) are used to validate the model’s effectiveness. Thus, SVM proves to be a powerful tool in polymer science, facilitating the rapid screening of polymer materials, optimization of formulations, and accurate prediction of new properties.

## 3. Tools for Implementing SVM in Polymer Science

The implementation of Support Vector Machine (SVM) algorithms in polymer science and engineering can be achieved through various software packages and libraries. These tools provide a user-friendly interface, optimized computational methods, and robust functionalities tailored for handling the complexities of polymer data, including nonlinearities, sparse data, and high-dimensional feature spaces.

1. One of the most widely used libraries for machine learning, Scikit-learn provides efficient and flexible tools to implement SVM models [22]. It offers a variety of functionalities, including SVM for classification and regression, kernel customization, parameter tuning (such as C and gamma), and model evaluation. With an easy-to-use API, Scikit-learn is highly popular among researchers working with polymer data due to its versatility, broad support, and compatibility with other scientific Python 3.x libraries such as NumPy, SciPy, and pandas.
2. LIBSVM [23] is a robust library specifically designed for SVM applications. It supports both classification and regression tasks, and its versatility in kernel functions (linear, polynomial, Gaussian radial basis function, etc.) makes it suitable for solving complex nonlinear problems often encountered in polymer science. LIBSVM has bindings for various programming languages such as Python, C++, Java, and MATLAB, making it flexible for integration into different environments. Its speed and accuracy are key reasons for its widespread adoption in both academic and industrial research.
3. MATLAB is commonly used in the field of polymer science for its strong computational power and ease of use for complex mathematical modeling. The Statistics and Machine Learning Toolbox in MATLAB includes a set of pre-built functions for implementing SVM [24] for classification, regression, and outlier detection. MATLAB’s visualization capabilities also allow researchers to graphically interpret the results of SVM models, making it useful for analyzing high-dimensional polymer datasets.
4. The e1071 package [25] in R provides tools for SVM implementation [26], specifically designed for classification, regression, and density estimation tasks. Researchers working with polymer data can benefit from R’s extensive data manipulation libraries, such as dplyr and ggplot2, for data preprocessing and result visualization. The e1071 package is known for its ease of use and accessibility, making it a popular choice for researchers conducting exploratory analysis of polymer properties.
5. WEKA (Waikato Environment for Knowledge Analysis) is a data mining software suite written in Java. It contains a variety of machine learning algorithms, including SVM [27], that are useful for classification and regression tasks. The user-friendly graphical interface and extensive documentation make it accessible to researchers without deep programming expertise. WEKA is widely used for educational purposes and is also employed in applied polymer science research to predict material properties and optimize processes.
6. While TensorFlow [28] and Keras [29] are more commonly associated with deep learning, they also offer functionality for traditional machine learning models, including SVM [30]. With these libraries, researchers can implement complex SVM models with large datasets, taking advantage of GPU acceleration for faster computation. TensorFlow’s compatibility with other tools, such as TensorFlow Extended (TFX) for production pipelines, is useful for polymer research requiring scalable and robust machine learning solutions.
7. SVMlight [31] is another widely used library that provides a fast and efficient implementation of SVM. It supports both classification and regression and is known for its speed and memory efficiency. This makes it suitable for large-scale datasets typical in polymer science. The tool also allows researchers to experiment with different kernel functions and optimization techniques to improve model performance.
8. KNIME [32] is a data analytics, reporting, and integration platform that integrates several machine learning algorithms, including SVM, into its workflow. Researchers in polymer science can use KNIME’s drag-and-drop interface to build end-to-end machine learning workflows, starting from data preprocessing to model evaluation. KNIME’s compatibility with other tools, such as Python and R, makes it a powerful platform for interdisciplinary research.

**Figure 9:**
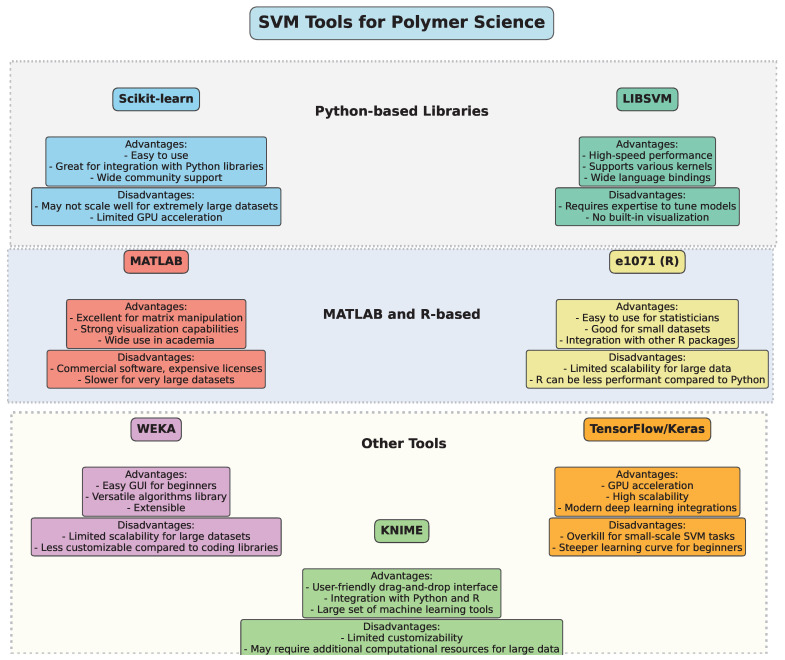
Overview of SVM tools.

Figure 9 provides an overview of several popular SVM tools, categorizing them by their programming environments and highlighting key advantages and disadvantages. This comparative diagram aims to guide researchers in choosing the optimal software tool based on factors like computational efficiency, ease of use, and integration with other machine learning methods, ensuring the best fit for their polymer science projects.

## 4. Case Studies

Han et al. [33] explore the use of three black-box modeling methods—SVMs, partial least squares (PLS) [34], and artificial neural networks (ANNs) [35]—to predict the melt index in two industrial polymerization processes: styrene–acrylonitrile [36,37] (SAN) and polypropylene [38,39] (PP). SVM models demonstrated the best prediction accuracy for both processes, outperforming PLS and ANN models. While SVMs were effective even with insufficient data or strong nonlinearities, ANNs struggled to predict accurately when data were sparse, especially in the PP process. PLS models, meanwhile, were less effective in the SAN process due to its strong nonlinear relationship with the melt index. The study also outlines the operational procedures for training and validating these models. The results indicate that SVMs are a reliable alternative for predicting the melt index in polymerization processes when dealing with nonlinearities.

Lee et al. [40] propose a modified SVM for estimating product properties in polymerization processes, which often involve high-dimensional, nonlinear, and sparse data. To address these challenges, the authors integrate locally weighted regression (LWR [41]) into the standard SVM, enhancing its ability to handle irregularly spaced data. The modified method assigns varying importance to training data based on their proximity to new input points, improving prediction accuracy for nonlinear process data. Case studies show that the weighted SVM (w-SVM) outperforms traditional SVR when dealing with high-dimensional and sparse datasets. The method was tested using two datasets: one from a polymer test plant and another from a polyvinyl butyrate (PVB) process. In both cases, the w-SVM demonstrated better predictive performance compared to standard SVM and other ML techniques. The results highlight the robustness of the w-SVM in improving estimation accuracy for nonlinear processes with irregular datasets. The study concludes that w-SVM is a promising approach for modeling and optimizing complex polymerization processes.

The core structure of polymer particles has an impact on optimizing hollow carbon nanospheres (HCNs). In the research by Yang et al. [42], pattern recognition methods were used to control and enhance the core polymer structure. A novel material design method based on a differential evolution SVM [43,44] (DE-SVM) was introduced, establishing a control model that was simulated digitally. The model helped identify the optimal conditions for controlling the pore structure of composite polymers, yielding better results than other classification methods. Experimental results demonstrated improved properties in hollow carbon spheres. In the experiment, PMMA core/shell polymer particles were carbonized to create HCNs, with the polymerization process influencing particle size and distribution. The study also employed an improved differential evolution algorithm for parameter optimization in support vector classification. This approach improved the classification accuracy and efficiency for identifying optimal structures for HCNs.

Mallakpour et al. [45] propose using quantitative structure–property relationships (QSPRs) to calculate the temperature of 10% mass loss (T10) for 50 optically active polymers. Descriptors were calculated from the repeating unit structures and selected using a genetic algorithm–PLS (GA–PLS) technique. The selected descriptors were then applied to an SVM model. The root mean square errors for the SVM model were smaller than those for the PLS model, indicating better predictive ability. The dataset was divided into training and prediction sets, with T10 data obtained under controlled conditions. Descriptors were generated from molecular structures using software like Hyperchem and Mopac, and Dragon for descriptor calculations. GA–PLS was used for variable selection, optimizing the model for accuracy with minimal variables. The study also evaluated the diversity of the compounds to ensure the robustness and predictive capability of the model.

Ziaee et al. [46] focuse on the implementation of the SVM algorithm to predict the solubility of CO2 in five different polymers: polystyrene (PS), polyvinyl acetate (PVAC), polypropylene (PP), polybutylene succinate-co-adipate (PBSA), and polybutylene succinate (PBS). The developed models demonstrate excellent agreement with experimental data, with average absolute relative deviations (%AARD) as low as 0.151% for PS and R^2^ values greater than 0.999. Additionally, the models’ performance is compared with traditional equations of state (EOS), artificial neural networks (ANNs), and adaptive neuro-fuzzy inference systems (ANFISs), showing superior robustness and efficiency. The paper also introduces Least Squares SVM [47] (LSSVM), which offer a computationally efficient alternative to traditional SVMs by employing linear equations for optimization instead of quadratic programming. Using kernel functions such as radial basis function (RBF) and polynomial kernels, LSSVMs provide accurate regression results for the studied polymers. The optimization of kernel parameters through coupled simulated annealing (CSA) [48] ensures optimal model performance. Furthermore, the study investigates potential outliers in the dataset and confirms the validity of the applied experimental data. Finally, the LSSVM models outperform other models in terms of accuracy, offering a reliable and effective method for predicting CO2 solubility in polymers, thus facilitating the analysis and design of polymer processing technologies.

Meng et al. [49] focuse on virtual screening of semiconductor polymers for high-performance organic photovoltaic (OPV) devices using ML algorithms, with particular emphasis on SVM and ensemble learning techniques. The study reveals that power conversion efficiency (PCE) can be predicted from the polymer structure fingerprints alone, without needing prior material property data. The predictive accuracy improves when results from SVM and random forest models are blended together, offering a more precise screening method. The dataset used in this research consisted of 1203 polymers, whose chemical structures were converted into SMILES codes to generate molecular fingerprints. The models were trained using these fingerprints to predict the PCE, with the SVM model yielding an average correlation coefficient (R) of 0.633. By integrating SVM and random forest models into an ensemble approach, the predictive accuracy was further enhanced, resulting in an R value of 0.653. This method was applied to a dataset of 316 polymers, from which high-performing compounds were identified, including one proposed structure with even higher predicted PCE. The study highlights the importance of refining data quality and addressing experimental variations to improve prediction accuracy for the virtual screening of OPV materials.

Vahid et al. [50] evaluate the capability of laser-induced breakdown spectroscopy (LIBS) combined with an SVM model for distinguishing polyvinyl chloride (PVC) from other polymers in recycling processes. A single-shot LIBS spectrum was recorded for each sample to ensure a rapid and practical technique in a recycling factory environment. Plasma emissions from five polymers—PE, PP, PS, PMMA, and PVC—were analyzed. The SVM used C2/C and N/C intensity ratios as input variables, achieving an accuracy of 90.5% using a polynomial kernel function of degree 2. The study demonstrates that LIBS, coupled with the nonlinear SVM, offers a fast and accurate classification method for polymer separation in recycling. LIBS spectra revealed key atomic and molecular lines, including C, N, and metallic elements, essential for polymer identification. The results suggest that further refinement, such as increasing the number of spectra, could improve accuracy.

Chen [51] demonstrates the use of SVM and ensemble learning to predict the power conversion efficiency (PCE) [52] of semiconductor polymers for organic photovoltaic (OPV) devices. The predictions were based solely on the polymers’ chemical structures, without requiring additional material properties. The chemical structures were represented using Morgan fingerprints derived from SMILES codes, and a dataset of 1203 polymers with corresponding PCE values was used. The SVM model, after optimizing the fingerprint radius, achieved a correlation coefficient of 0.633, while the random forest (RF) model showed a slightly higher R value of 0.640. Combining the predictions from SVM and RF through ensemble learning resulted in an improved correlation coefficient of 0.653. This method shows potential for high-throughput virtual screening of OPV polymers. However, the study acknowledges challenges due to the sensitivity of PCE to experimental conditions, and enhancing data quality could improve predictive accuracy. Overall, this work contributes to advancing material design using artificial intelligence.

Zhu et al. [53] present a system for identifying plastic solid waste (PSW) using near-infrared (NIR) reflectance spectroscopy combined with an SVM classification model. A device was developed to obtain the NIR spectra of plastics, and spectral preprocessing techniques such as normalization and smoothing were applied to improve spectral repeatability. The study introduced a PCAeSVM identification method, achieving 97.5% accuracy in identifying six types of plastics, including polypropylene (PP), polystyrene (PS), polyethylene (PE), poly(methyl methacrylate) (PMMA), acrylonitrile butadiene styrene (ABS), and polyethylene terephthalate (PET). The system can distinguish plastic types and discern sample shapes, with potential application in industrial recycling. A total of 186 samples were used to build the identification model and validate the results. The system demonstrated strong classification ability with a 97.5% accuracy, although some plastic types with similar spectral features were misidentified. Improvements suggested include adding multiple NIR probes to enhance spectral collection efficiency and building a comprehensive NIR polymer database to expand the system’s capabilities.

Tokuyama et al. [54] examine the lower critical solution temperature (LCST) behavior of a thermosensitive NIPA-co-MTGA polymer in aqueous salt solutions. The LCST of the NIPA-co-MTGA polymer was found to be between that of the NIPA and MTGA homopolymers, and it varied with the polymer composition. The LCST decreased with increasing salt concentration, and the extent of this decrease depended on the type of salt, following the Hofmeister series. The LCST was successfully predicted using SVR, marking the first time SVR has been applied to predict the LCST of thermosensitive polymers in salt solutions. The model showed strong predictive ability, with good agreement between experimental and predicted LCST values for various salts. Additionally, the SVR model outperformed multiple linear regression (MLR) in prediction accuracy. These findings highlight the potential of ML approaches in understanding and predicting the behavior of thermosensitive polymers. This work opens avenues for future studies applying ML to polymer research.

Particle swarm optimization-based SVR [55] (PSVR) and Ordinary Linear Regression (OLR) models were developed by Owolabi et al. [56] to estimate the refractive index (n) and energy gap (E) of polyvinyl alcohol composites. The n-PSVR model, using the energy gap to predict the refractive index, outperformed the n-OLR model in terms of root mean square error (RMSE) and mean absolute error (MAE). Similarly, the E-PSVR model, predicting the energy gap based on the refractive index, showed better performance than the E-OLR model. These models were employed to investigate the effects of sodium-based dysprosium oxide and benzoxazinone derivatives on the energy gaps of polyvinyl alcohol composites. The results demonstrated a high degree of accuracy, with the models aligning well with experimental data. Validation with external data further confirmed their reliability, yielding low mean absolute percentage errors. These findings indicate that the models can effectively reduce experimental costs while optimizing the optical properties of polyvinyl alcohol composites. The computational methodology involved optimizing hyperparameters using particle swarm optimization, ensuring the precision and robustness of the models.

The aging of transformer polymer insulation is often monitored by analyzing various aging indicators, with a focus on the concentration of dissolved gases in oil. Recent studies have highlighted alcohols like methanol and ethanol as new aging indicators, yet there is limited research on predicting their concentrations in transformer oil. To address this gap, Wu et al. [57] present a genetic-algorithm-optimized SVM [58] (GA-SVM) model for predicting methanol and ethanol concentrations. The study involves accelerated thermal aging experiments to collect data on alcohol concentrations, using four days of historical data to predict future concentrations. GA-SVM optimizes the kernel function parameter and penalty factor of the SVM to enhance prediction accuracy. The model’s predictions were compared with those from other algorithms, showing that GA-SVM outperforms the alternatives. The results demonstrated an MSE of 0.008 for methanol and 0.003 for ethanol. The study also proposes a software tool for industrial applications to predict these alcohol concentrations based on this model. The research also emphasizes the importance of optimizing SVM parameters using genetic algorithms, with the penalty factor C and kernel parameter g being key to the model’s predictive power. Using fivefold cross-validation, the model was fine-tuned, achieving the best predictive results. The GA-SVM model’s effectiveness was validated by comparing its performance to other intelligent algorithms, showing its superiority in predicting methanol and ethanol concentrations in transformer oil.

Nie et al. [59] propose a method to identify different colored plastics using laser-induced breakdown spectroscopy (LIBS) combined with neighborhood component analysis (NCA) and SVM algorithms. The method was tested on six types of plastics (PVC, POM, ABS, PP, PA, and PE) with various colors, showing an average accuracy of over 97% for plastic type identification. When the same types of plastics with different colors were classified, the accuracy improved to over 99%. However, PVC identification accuracy was only 82%, which was improved to 91% by incorporating NCA for feature selection. NCA helps enhance the accuracy by selecting spectral lines with higher feature weights, reducing dimensionality. Compared to SVM and PCA-SVM, the NCA-SVM model improved performance by identifying PVC samples with higher precision. This combined approach of LIBS with SVM and NCA demonstrates strong potential for efficient plastic recycling through high-accuracy identification of both plastic types and colors.

Sumayli et al. [60] proposed a computational methodology by combining computational fluid dynamics (CFD) and ML for simulating mass transfer in membrane systems. The approach utilized CFD data to train ML models, with a case study involving a polymeric membrane for solute removal. The dataset contained over 8000 data points, with two input coordinates (r, z) and one output, solute concentration (C). The AdaBoost algorithm was employed with three base models: decision trees (DTs), Theil–Sen Regressions (TS), and SVR. Among these models, the Boosted Decision Tree exhibited the highest accuracy, with an R^2^ of 0.9978, a root mean square error (RMSE) of 39.6, and a mean absolute error (MAE) of 10.1. The model’s performance was further validated through 3D plots, illustrating the solute concentration’s decline along the axial direction (z) of the membrane. This methodology holds potential for optimizing membrane separation processes by enhancing concentration decay, which improves separation efficiency.

SVR was utilized by Uddin et al. [61] to predict the glass transition temperature ($T_{g}$) of polymers, focusing on the relationship between molecular descriptors and $T_{g}$. The dataset consisted of 7174 samples, and SVR’s performance was compared with other algorithms such as Extra Tree Regressor (ETR) and random forest (RF). Among the models tested, SVR achieved an R^2^ value of 0.684, MAE of 47.604, and RMSE of 62.467, demonstrating moderate accuracy in $T_{g}$ prediction. Feature selection techniques, including Pearson correlation and recursive feature elimination, were applied to improve the model’s performance. Despite its moderate performance, SVR’s ability to handle high-dimensional data and its robustness to overfitting [62] make it a valuable tool for predicting $T_{g}$ in polymer science. Additionally, the SHAP method was used to interpret the impact of molecular descriptors on the model’s output. The results indicate that SVR can serve as a useful model for polymer design, but further optimization may enhance its accuracy in practical applications.

Zeolite has shown promise as an additive for improving the hydrophilicity of polymeric membranes, but its role in enhancing mechanical strength has been limited. Chan et al. [63] explore the modification of poly(vinylidene fluoride) (PVDF) membranes by incorporating zeolite at concentrations ranging from 0.5 to 2 wt% to improve both mechanical properties and filtration performance. The membranes were fabricated using a dry–wet phase inversion technique, and their morphological structure, mechanical strength, and hydrophilicity were analyzed. Incorporating 0.5 wt% zeolite increased the tensile strength to 19.4 MPa and enhanced the membrane’s hydrophilicity, resulting in a 63.49% increase in pure water flux and 95.76% BSA rejection compared to pristine PVDF membranes. Field-emission scanning electron microscopy [64] (FESEM) revealed dense, microvoid-free structures in all PVDF membranes, with the zeolite-modified versions showing denser skin layers. Additionally, the SVR model predicted the molecular weight cut-off (MWCO) of the membranes with high accuracy (R^2^ = 0.855). The findings highlight the potential of zeolite to enhance both the mechanical properties and separation performance of PVDF membranes for ultrafiltration applications.

Amer et al. [65] explore the prediction of ultimate strain in anchored carbon fibre-reinforced polymer (CFRP) laminates [66], which is crucial for assessing the flexural strength of concrete beams strengthened with CFRP. Various parameters, including CFRP sheet width, anchor design details, and the number of CFRP layers, were examined. ML models, such as SVR and decision trees, were tested, with linear regression emerging as the most accurate. The study develops a predictive equation for ultimate strain, derived from statistical regression analysis. Additionally, the study includes an extensive dataset of concrete prisms, where reliability and correlation are thoroughly analyzed. The proposed equation offers practical recommendations for design optimization, highlighting the importance of anchor parameters in enhancing CFRP laminate performance.

Table 1 provides a summary of key studies that have been reviewed in this paper. The table highlights the main focus of each study, the specific SVM models applied, the key findings, and any noted limitations.

## 5. Limitations and Future Work

**Figure 10:**
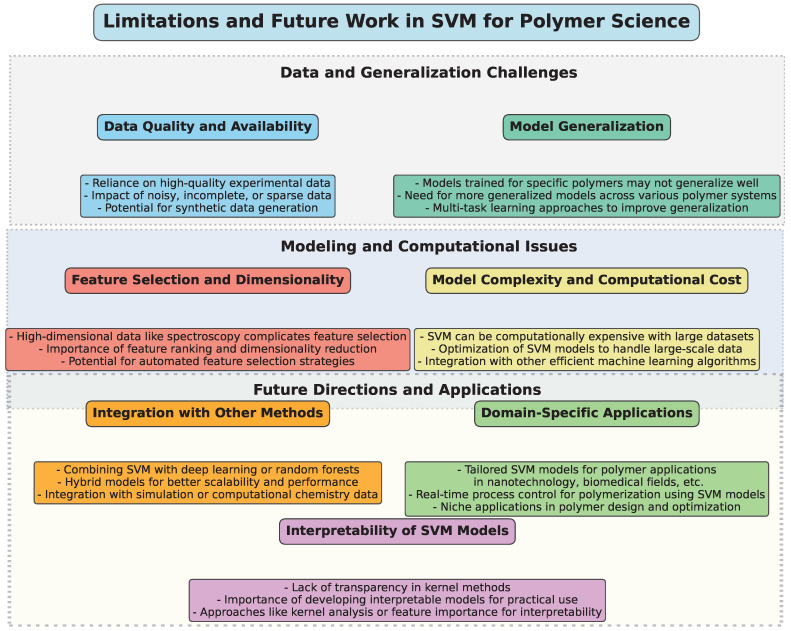
Illustrative diagram summarizing the limitations and future directions in the application of SVM in polymer science.

To provide a structured overview of the challenges, limitations, and potential future directions for the application of SVM in polymer science, a diagram (Figure 10) has been constructed. This visual representation categorizes key aspects into thematic groups, highlighting the interconnected nature of these issues.

SVM has proven to be an effective and versatile tool in polymer science, particularly for predicting material properties, identifying polymer types, and optimizing polymerization processes. However, despite the widespread application of SVM, several limitations and challenges continue to arise, highlighting the need for further research and development in this area.

A key limitation of SVM models, especially in polymer science, is the reliance on high-quality experimental data. As highlighted in multiple studies, SVM’s performance is highly sensitive to the quality and quantity of the data available. Inaccurate, incomplete, or sparse data can significantly reduce the predictive accuracy of SVM models, as seen in studies such as those by Han et al. [33] and Mallakpour et al. [45], where sparse datasets impacted the performance of SVM. Future work should focus on improving data acquisition methods and developing robust models that can handle noisy and incomplete data effectively. Additionally, researchers should explore methods to generate synthetic datasets using techniques like data augmentation or transfer learning, when experimental data are limited.

Many studies have demonstrated that SVM can provide accurate predictions for specific polymers or polymer systems, such as in the case of predicting polymer core structures [42] or solubility [46]. However, the transferability of models across different types of polymers remains a challenge. Models trained on one specific polymer often fail to generalize well to other polymers with different characteristics, as observed in the research by Tokuyama et al. [54] and Vahid et al. [50]. Therefore, a promising direction for future work is the development of more generalized SVM models capable of accurately predicting properties for a broad range of polymers, or the incorporation of multi-task learning approaches that can account for a wider variety of polymer types simultaneously.

Another common challenge is selecting the most relevant features for SVM models, when dealing with high-dimensional data, such as spectral or chemical property data. In many polymer studies, like those using near-infrared spectroscopy (NIR) [53], the data can be high-dimensional, which complicates the selection of the most informative features. This can lead to overfitting or underfitting, thereby reducing the reliability of predictions. Researchers are working on more sophisticated feature selection techniques, including feature ranking methods and dimensionality reduction techniques like Principal Component Analysis (PCA). Future research may focus on integrating feature selection strategies with SVM to enhance their performance when handling complex, multi-dimensional datasets.

While SVM is known for its strong performance in classification and regression tasks, it can be computationally expensive, especially with large and complex datasets. For example, in polymer recycling or large-scale polymer synthesis models, the computational cost can quickly become prohibitive when using nonlinear kernels or hyperparameter optimization techniques. Future work may explore the development of more efficient SVM algorithms, such as those that implement approximation techniques or those that reduce the need for exhaustive cross-validation during training. Hybrid approaches combining SVM with other ML models, such as deep learning or ensemble methods, may also offer solutions for scaling up models while maintaining efficiency.

Interpretability [67,68] is a significant concern, especially when applying SVM models to real-world polymer science problems. In applications like material design or process optimization, it is crucial for researchers to understand the underlying reasons behind the model’s predictions. While kernel methods enhance SVM’s performance, they also introduce a level of complexity that makes the model less transparent. Recent work, such as that by Sumayli et al. [60], has explored boosting interpretability through methods like kernel analysis or feature importance scores. However, more attention should be given to developing techniques that provide clearer insights into how SVM models arrive at their decisions, thereby enhancing their utility in decision-making processes.

Another promising avenue for future work involves combining SVM with other ML techniques, such as deep learning, random forests, or genetic algorithms [69,70,71]. Studies have shown that ensemble methods, such as the hybrid use of SVM with random forests or decision trees, can improve predictive performance, as seen in the work by Meng et al. [49]. Integrating SVM with optimization algorithms like genetic algorithms or particle swarm optimization (PSO) could also lead to improvements in polymer property prediction and polymerization process control. Moreover, multi-modal approaches that combine SVM with spectroscopy, computational chemistry, and simulation data could provide more holistic models for polymer-related applications.

While SVM has been applied to a wide range of polymer science applications, there are still many domain-specific challenges that require tailored approaches. For example, polymeric materials used in nanotechnology, biomedical applications, or energy storage require models that account for unique material properties and behavior. Furthermore, real-time process monitoring and control in polymerization processes, such as in the work by Lee et al. [40], would benefit from SVM models integrated with real-time data acquisition systems. Future research could explore these niche areas and develop specialized models to address the unique needs of these industries.

## 6. Conclusions

Han et al. [33] investigate the application of black-box modeling methods—SVMs, and ANNs—to predict the melt index in polymerization processes for styrene–acrylonitrile (SAN) and polypropylene (PP). The study found that Support Vector Machines (SVMs) outperformed both PLS and ANN models in cases with insufficient data or strong nonlinearities, highlighting SVM’s robustness. ANN models, while effective in some contexts, struggled with sparse data in the PP process, while PLS models were less effective due to the complex nonlinearity in the SAN process. These findings underscore the advantages of SVM models for predicting the melt index in polymerization processes, where nonlinear relationships are common.

To further enhance SVM’s performance in such complex scenarios, integrating ensemble methods, such as a hybrid SVM-ANN, could help overcome issues with sparse data in ANN-based approaches. Moreover, implementing a more dynamic and adaptive selection of kernel functions in SVM could improve its robustness in diverse polymer processes, adapting to varying degrees of nonlinearity.

Lee et al. [40] proposed an innovative approach combining locally weighted regression (LWR) with SVM to estimate product properties in polymerization processes that often involve high-dimensional and nonlinear data. The weighted SVM (w-SVM) improved prediction accuracy, particularly for irregularly spaced data. Testing with datasets from a polymer test plant and polyvinyl butyrate (PVB) process, w-SVM outperformed traditional SVM models and other machine learning techniques. This reinforces the robustness of w-SVM in predicting complex polymerization behaviors and suggests its promise for modeling and optimizing polymerization processes.

While w-SVM improves upon traditional SVM, a potential enhancement could involve the development of an adaptive weighting mechanism. For example, introducing a time-series-dependent weighting mechanism could be beneficial for processes that evolve over time. This would allow the model to adapt more effectively to new data patterns during the polymerization process, improving both prediction accuracy and process control.

A different area of polymer process optimization, Yang et al. [42] explored using pattern recognition and differential evolution SVM (DE-SVM) to improve the core structure of hollow carbon nanospheres (HCNs). Their approach helped optimize the polymerization conditions for PMMA core/shell polymer particles, improving the distribution of the carbonized HCNs. Experimental results confirmed that DE-SVM could efficiently identify optimal polymer structures, outperforming traditional methods. This work demonstrates how SVM-based modeling can be leveraged for novel material design in advanced polymer applications.

To enhance the DE-SVM approach, incorporating a multi-objective framework could provide more insights into trade-offs between various properties (e.g., mechanical strength vs. porosity). Additionally, using hybrid algorithms like genetic algorithms combined with DE-SVM could allow the model to more efficiently explore and identify optimal solution spaces, especially for more complex multi-phase polymerization processes.

In the realm of predictive modeling of polymer properties, Mallakpour et al. [45] employed quantitative structure–property relationships (QSPRs) to predict the temperature of 10% mass loss (T10) for a range of optically active polymers. Using SVM with descriptors optimized through a genetic algorithm and PLS for variable selection, they achieved better predictive accuracy than PLS alone. This study illustrates the powerful combination of molecular structure descriptors and machine learning models like SVM in accurately predicting polymer thermal stability.

One potential improvement would be the integration of feature engineering techniques, such as Principal Component Analysis (PCA), before applying SVM to reduce multicollinearity and further improve model performance. Additionally, the incorporation of deep learning techniques such as autoencodersfor feature extraction could uncover hidden patterns in the structural descriptors, leading to even more accurate predictions.

Ziaee et al. [46] focused on predicting CO2 solubility in polymers using SVM and Least Squares SVM (LSSVM) models, comparing their performance to traditional methods like ANN and adaptive neuro-fuzzy inference systems (ANFISs). Their LSSVM model achieved excellent predictive performance, with minimal error and a high correlation with experimental data, outpacing the other models in both robustness and efficiency. This work highlights the utility of SVM-based models in predicting solubility and improving the understanding of polymer properties for industrial applications.

A potential improvement for LSSVM would involve exploring a multi-kernel strategy, where different kernels are applied based on the nature of the data. For example, using radial basis function (RBF) kernels for smooth data and polynomial kernels for more complex relationships can improve the generalization ability of LSSVM, making it even more robust for different types of solubility predictions.

Meng et al. [49] applied SVM and ensemble learning techniques, such as random forests, for the virtual screening of semiconductor polymers used in organic photovoltaic (OPV) devices. By predicting power conversion efficiency (PCE) from polymer structure alone, their SVM model provided a solid foundation for screening high-performance materials, with improvements achieved through ensemble methods. This study suggests that SVM can effectively predict performance from chemical fingerprints, advancing the field of materials discovery in OPV research.

One enhancement could involve incorporating transfer learning methods, where a model trained on one set of materials (e.g., small molecules) is adapted to predict properties of polymers. This approach can be useful in reducing the need for large datasets and overcoming the challenge of limited data availability in polymer material discovery.

In the recycling and polymer identification domain, Vahid et al. [50] utilized laser-induced breakdown spectroscopy (LIBS) combined with SVM for fast and accurate polymer classification in recycling processes. Their method, using intensity ratios as input variables, achieved over 90% accuracy in distinguishing different polymers, including PE, PP, PS, PMMA, and PVC. This study highlights the efficiency of combining LIBS and SVM for high-speed identification in industrial settings, offering practical benefits for material sorting in recycling.

Chen [51] used SVM and ensemble learning to predict the power conversion efficiency of OPV polymers, relying solely on polymer chemical structures. By utilizing molecular fingerprints and combining predictions from SVM and random forests, the study achieved a modest improvement in predictive accuracy.

One potential avenue for improvement is the incorporation of molecular dynamics simulations to generate more accurate and detailed molecular descriptors. Coupled with machine learning models, this could allow for a more comprehensive understanding of the structure–property relationships in OPVs, enabling the design of polymers with superior performance.

Zhu et al. [53] investigated plastic solid waste identification using near-infrared (NIR) spectroscopy coupled with SVM. Their approach achieved an impressive 97.5% accuracy in identifying six types of plastics, demonstrating the effectiveness of NIR spectroscopy in recycling applications. The incorporation of multi-modal sensing techniques, such as hyperspectral imaging or multispectral sensing, could provide more robust and detailed identification capabilities. Additionally, leveraging active learning to continuously update the model with new data could help improve accuracy in dynamic recycling environments, where new polymer blends are frequently introduced.

In predicting the behavior of thermosensitive polymers, Tokuyama et al. [54] used Support Vector Regression (SVR) to predict the lower critical solution temperature (LCST) of a thermosensitive NIPA-co-MTGA polymer in salt solutions. The study found that SVR provided strong predictions, outperforming multiple linear regression. To enhance SVR for predicting the LCST, one could explore the use of a kernelized feature selection method, which could help in identifying the most relevant factors affecting LCST behavior, thereby improving prediction accuracy. Moreover, incorporating multi-task learning could help in predicting the LCST of various thermosensitive polymers simultaneously, increasing the efficiency of model training.

Owolabi [56] utilized particle swarm optimization-based SVR (PSVR) to predict the refractive index and energy gap of polyvinyl alcohol composites. Their models showed improved performance over traditional regression techniques. A potential enhancement would be to implement a hybrid approach, combining PSVR with deep reinforcement learning (DRL). By incorporating DRL, the model could adaptively tune hyperparameters during training, optimizing predictions based on past performance and reducing the need for manual fine-tuning.

Wu et al. [57] developed a GA-SVM model to predict the concentration of methanol and ethanol in transformer oil, demonstrating its superiority over other algorithms. To further improve the model’s accuracy, one could integrate a data augmentation strategy to generate synthetic data for training, especially for rare or extreme events. This could help to make the model more robust in predicting real-world scenarios where data might be limited or skewed.

Nie et al [59] extended the application of SVM for identifying different colored plastics using LIBS, achieving an accuracy of over 97%. Leveraging transfer learning could be beneficial in handling plastic identification across different recycling plants or regions. By pre-training the model on one dataset and then fine-tuning it on local data, the system could adapt to regional variations in plastic materials, improving both accuracy and generalizability.

Sumayli et al. [60] explored the integration of CFD with machine learning to simulate mass transfer in membrane systems. The addition of uncertainty quantification to the CFD–ML modelscould help in evaluating the reliability of the predictions, especially when applied to real-world scenarios where there is variability in the operating conditions of membrane systems.

Finally, Uddin et al. [61] applied SVR to predict the glass transition temperature (Tg) of polymers, demonstrating the model’s capability despite moderate performance compared to other algorithms. Incorporating explainability into the SVR modelcould help users understand how specific features influence Tg prediction. Techniques like SHAP (Shapley Additive Explanations) could be employed to increase transparency and guide researchers in identifying the key factors affecting Tg, improving the model’s usability.

The main contribution of this study can be summarized as follows:

- SVM outperformed other machine learning techniques such as PLS and ANN in predicting key properties like the melt index, CO2 solubility, and power conversion efficiency in polymer systems
- Handling Nonlinear and Sparse Data: The integration of weighted regression (w-SVM) and differential evolution SVM (DE-SVM) enhanced the handling of sparse and nonlinear data, improving prediction accuracy in polymerization processes
- SVM was successfully applied across a variety of tasks, including predicting semiconductor polymer properties, estimating CO2 solubility, and identifying plastic types for recycling
- Optimization of kernel parameters, such as radial basis and polynomial kernels, improved SVM’s performance, with techniques like coupled simulated annealing (CSA) and genetic algorithms further enhancing model accuracy
- SVM contributed to the optimization of hollow carbon nanospheres (HCNs) and polymer composites by identifying the optimal conditions for improved material properties
- SVM consistently outperformed other methods, such as ANNs and random forests, particularly in terms of accuracy and efficiency for high-dimensional datasets
- In industrial applications like plastic identification and transformer insulation monitoring, SVM models proved effective in improving accuracy and predictive power
- While SVM has proven effective, challenges related to data quality, outliers, and model interpretation remain. Future research should focus on optimizing feature selection, integrating ensemble methods, and improving accuracy.
- SVM-based approaches have great potential in polymer recycling, demonstrating high accuracy in plastic type identification.

In summary, the reviewed studies collectively emphasize the growing potential of Support Vector Machines (SVMs) and other machine learning techniques across various domains of polymer research, including process optimization, material design, property prediction, and recycling. While SVM consistently demonstrates strong predictive capabilities, challenges related to data sparsity, nonlinearity, and experimental variations remain. Future research should focus on optimizing machine learning models, improving data quality, and expanding their application to other complex polymer behaviors.

## Figures and Tables

**Table 1:** Summary table of SVM applications in polymer science.

Reference	Focus	Applied Model	Results	Limitations
Han et al. [33]	Prediction of melt index in polymerization processes	SVM, PLS, ANN	SVM outperforms PLS and ANN, especially for nonlinearities.	ANN struggles with sparse data, PLS ineffective in SAN process.
Lee et al. [40]	Estimating product properties in polymerization processes	w-SVM	w-SVM outperforms traditional SVR in handling sparse and high-dimensional data.	Requires careful tuning for highly irregular datasets.
Yang et al. [42]	Control of polymer core structure for hollow carbon nanospheres	DE-SVM	DE-SVM improves core particle structure for HCNs.	Limited to the specific polymer studied.
Ziaee et al. [46]	Solubility of CO2 in different polymers	LSSVM	LSSVM offers superior performance compared to traditional EOS and other models.	Requires optimal kernel parameter tuning for best results.
Meng et al. [49]	Screening polymers for organic photovoltaic devices	SVM + random forest (ensemble)	Ensemble method improves predictive accuracy of power conversion efficiency (PCE).	Data quality variability affects model accuracy.
Vahid et al. [50]	Identifying PVC in recycling processes	LIBS + SVM	SVM with polynomial kernel achieves 90.5% accuracy for polymer classification.	Misidentification of plastics with similar spectra.
Chen [51]	Predicting PCE of semiconductor polymers for OPV devices	SVM + random forest (ensemble)	Ensemble improves prediction accuracy for PCE.	Experimental conditions affect prediction accuracy.
Zhu et al. [53]	Identifying plastic types using NIR spectroscopy	PCAeSVM	97.5% accuracy in classifying six plastic types.	Misidentification of plastics with similar spectral features.
Tokuyama et al. [54]	Predicting LCST of thermosensitive NIPA-co-MTGA polymer	SVR	SVR model shows strong predictive ability for LCST.	Limited to specific polymer and salt types.
Owolabi et al. [56]	Estimating refractive index and energy gap of polyvinyl alcohol composites	PSVR	PSVR models outperform OLR models with high accuracy.	Not applicable to all types of composites.
Wu et al. [57]	Predicting alcohol concentration in transformer oil	GA-SVM	GA-SVM outperforms other models, with MSE values for methanol and ethanol.	Relies on high-quality experimental data.
Nie et al. [59]	Identifying colored plastics using LIBS	LIBS + NCA + SVM	97% accuracy for plastic identification, with improved results for colored plastics.	PVC identification accuracy could be improved.
Sumayli et al. [60]	Simulating mass transfer in membrane systems	AdaBoost (with DT, TS, SVR)	Boosted Decision Tree offers high accuracy (R^2^ = 0.9978).	Further testing required for broader membrane systems.
Uddin et al. [61]	Predicting glass transition temperature ($T_{g}$) of polymers	SVR	SVR provides moderate accuracy for $T_{g}$ prediction.	Further optimization needed for higher accuracy.
Chan et al. [63]	Enhancing mechanical strength and filtration performance of PVDF membranes	SVR	SVR model predicts MWCO with high accuracy (R^2^ = 0.855).	Limited to specific types of membrane modifications.
Amer et al. [65]	Predicting ultimate strain in CFRP laminates	SVR	SVR provides accurate predictions for strain, with design optimization recommendations.	Model sensitivity to data quality.
Mallakpour et al. [45]	Predicting temperature for mass loss in optically active polymers	GA-PLS + SVM	SVM outperforms PLS, offering better prediction accuracy.	GA-PLS may be computationally expensive.
